# Rupture of fetal membrane in ectopic tubal pregnancy: A case report and literature review

**DOI:** 10.1097/MD.0000000000039713

**Published:** 2024-09-20

**Authors:** Zhiqiang Wang, Chun Zhang

**Affiliations:** a Department of Gynecology, The Central Hospital of Wuhan, Tongji Medical College, Huazhong University of Science and Technology, Wuhan, China.

**Keywords:** ectopic pregnancy, fallopian tube, fetal membrane

## Abstract

**Rationale:**

Ruptured tubal pregnancies occurring in the second trimester are rare; yet, they pose a critical risk of life-threatening hemorrhage. This study aims to highlight the importance of timely surgical intervention in such cases to prevent fatal outcomes. The case underscores the diagnostic and therapeutic challenges that arise when distinguishing between tubal and abdominal pregnancies, particularly in the presence of hemoperitoneum, which can obscure imaging results.

**Patient Concerns:**

We present a case involving the spontaneous rupture of a tubal pregnancy at 15 weeks and 3 days of gestation. The patient exhibited elevated beta-human chorionic gonadotropin levels. Initial transabdominal ultrasound suggested an abdominal pregnancy, and computed tomography scans supported these findings.

**Diagnoses and Interventions:**

Urgent midline laparotomy revealed the condition to be a tubal pregnancy, contrary to initial imaging. The surgical procedure included the removal of the gestational sac and the affected fallopian tube, followed by abdominal closure. Hemoperitoneum was noted to compromise the accuracy of imaging modalities, complicating the preoperative diagnosis.

**Outcomes:**

Histopathological examination confirmed the diagnosis of tubal pregnancy. The patient had an uneventful recovery and was discharged 7 days post-surgery with stable hemoglobin levels.

**Lessons:**

This case underscores the importance of considering the differential diagnosis of abdominal versus tubal pregnancy in the presence of hemoperitoneum, due to their differing clinical management needs. It offers insights that may guide clinicians in the timely diagnosis and treatment of advanced tubal pregnancies, where prompt surgical intervention is critical.

## 1. Introduction

Ectopic pregnancy, characterized by implantation of the blastocyst outside the endometrial lining of the uterine cavity, is a significant obstetric complication associated with maternal morbidity and mortality. Among ectopic pregnancies, tubal ectopic pregnancies are the most common, occurring when the blastocyst implants within the fallopian tube instead of the uterus. However, in rare instances, ectopic pregnancies can occur in other extrauterine locations, such as the abdomen, ovaries, cervix, or even within a previous cesarean scar.^[1,2]^

Here, we present a unique case of a 27-year-old primigravida with a history of prior abortions, who presented to the emergency department with acute lower abdominal pain, nausea, vomiting, and rectal pressure. Despite experiencing vaginal spotting following a previous episode of hemorrhage, the patient’s symptoms were initially attributed to menstrual irregularities, leading to delayed recognition of the ectopic pregnancy.

## 2. Case Report

A 27-year-old Chinese primigravida with a history of 2 abortions presented to the emergency department with acute lower abdominal pain for 2 hours, accompanied by nausea, vomiting, and a sensation of rectal pressure. Mistaking her hemorrhage a month prior to menstrual bleeding, she had been experiencing intermittent vaginal spotting since then. She found herself incapacitated by muscle weakness, necessitating ambulance transport to the hospital, where she arrived with stable vital signs. Condoms are her preferred method of contraception, and she has never used emergency contraceptives. She maintains good sexual hygiene, with no history of promiscuity, pelvic inflammatory disease, or endometriosis. Her medical records show no history of abdominal surgery, and she leads a healthy lifestyle, abstaining from alcohol and smoking. Her regular occupation involves light physical work. Additionally, she categorically denies any history of anemia or other chronic health issues.

**Figure 1.**
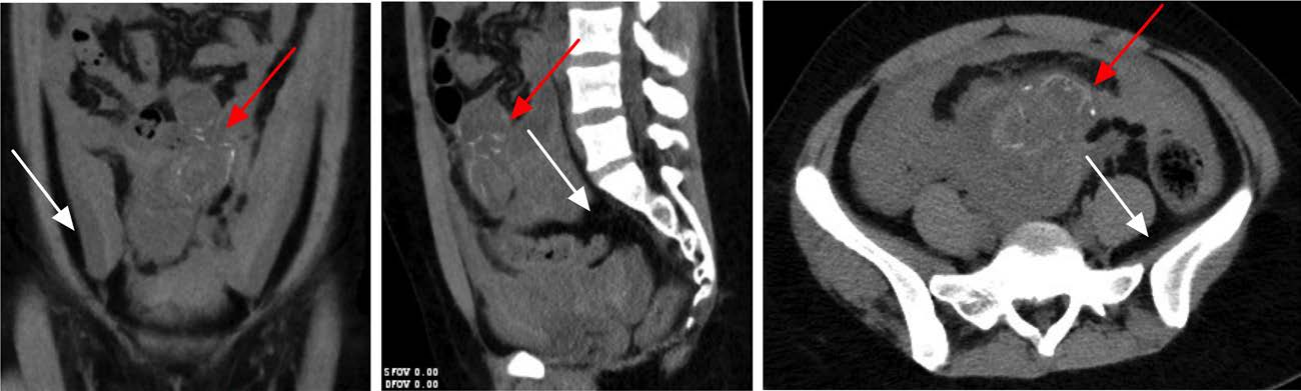
Preoperative computed tomography scan of the abdomen with arrow marks showing fetal mass (red) and bloody effusions (white).

**Figure 2.**
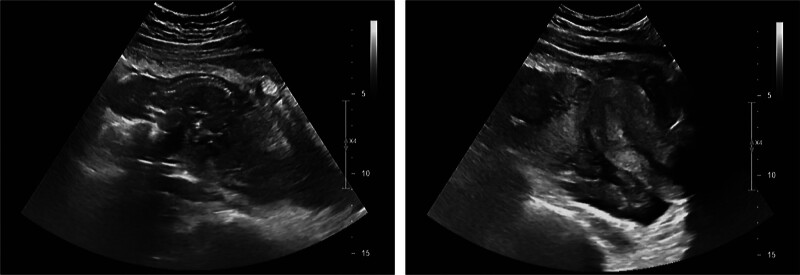
Transabdominal ultrasound of abdominal cavity showing the living fetus with a gestational age of 15 weeks and 3 days.

Upon examination, her abdomen was notably distended and tender, with signs of peritonitis. The absence of active vaginal bleeding or discharge was confirmed, although cervical motion and bilateral adnexal tenderness were also evident. Laboratory tests indicated significantly elevated serum β-hCG levels and leukocytosis, with a moderate decrease in hemoglobin levels. Computed tomography revealed a fetal mass and considerable intra-abdominal hemorrhage (Fig. 1). Ultrasonography failed to detect an intrauterine gestational sac, but showed a live mid-abdominal fetus corresponding to 15 weeks and 3 days of gestation, with a healthy fetal heart rate, suggesting an abdominal pregnancy (Fig. 2).

**Figure 3.**
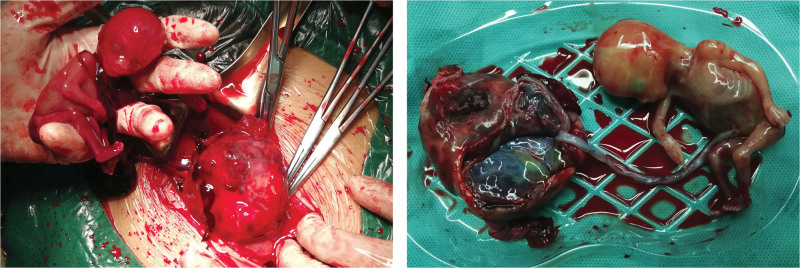
Images of ruptured tubal ectopic amniotic cavity with fetus and placenta.

**Figure 4.**
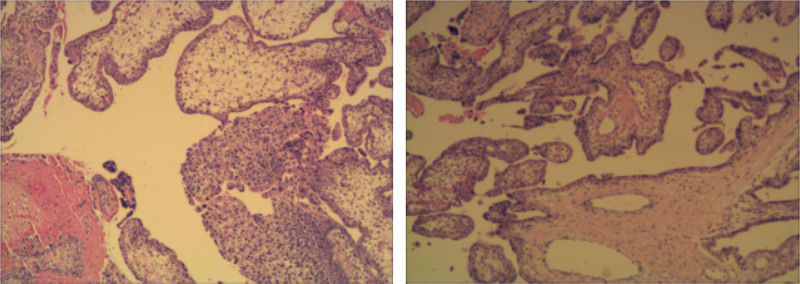
Microscopic histological sections further supported the diagnosis of tubal pregnancy which showed the wall of fallopian tube and a tubal pregnancy with decidua, chorionic villi, and syncytiotrophoblast inside the fallopian tube (×10).

Following initial stabilization and rapid preparation for surgery in the gynecological ward, her condition deteriorated, prompting immediate surgical intervention. During the laparotomy, extensive hemoperitoneum was encountered, and the ruptured ectopic pregnancy in the right fallopian tube was identified, resembling a “ground ball” in size, in contrast to the normal appearance of the left tube. The fetus was expelled into the abdomen, still attached to the placenta, with active bleeding noted at the rupture site (Fig. 3). Adhesion separation poses a risk of further bleeding and damage to adjacent organs. The surgery proceeded smoothly, successfully, and completely removing the affected fallopian tube and pregnancy tissue, followed by hemostasis treatment of the mesosalpinx wound. The operation necessitated substantial blood transfusion, including autotransfusion and excision of the affected tube for pathological examination, which later confirmed a well-developed placental lining (Fig. 4).

The patient underwent urgent midline laparotomy, during which a ruptured tubal pregnancy was identified, contrary to initial imaging that suggested an abdominal pregnancy. The surgical procedure included the removal of the gestational sac and the affected right fallopian tube, followed by hemostasis treatment of the mesosalpinx wound. Despite the presence of extensive hemoperitoneum, the operation was successful, and the patient required substantial blood transfusion, including autotransfusion of the collected blood during surgery.

Histopathological examination of the removed tissue confirmed the diagnosis of a well-developed placental lining within the ruptured fallopian tube, supporting the diagnosis of a tubal pregnancy. The patient experienced an uneventful recovery, with stable hemoglobin levels postoperatively. She was discharged from the hospital 7 days after the surgery in stable condition. This case was reviewed and approved by the Institutional Review Board of the Central Hospital of Wuhan (No. 2024-083-01) and informed consent from the patient was obtained for the publication of this case report.

This case highlights the importance of prompt surgical intervention in managing second-trimester tubal pregnancies, which, although rare, carry a high risk of severe hemorrhage. The findings emphasize the need for careful differential diagnosis when imaging results suggest an abdominal pregnancy, especially in the presence of hemoperitoneum. These outcomes provide critical insights for clinicians, particularly in the timely diagnosis and treatment of advanced ectopic pregnancies.

## 3. Discussion

In ectopic or extrauterine pregnancy, the blastocyst implants anywhere other than the endometrial lining of the uterine cavity.^[3]^ The presentation of extrauterine pregnancy varies, from an asymptomatic state to postmenopausal uterine bleeding, lower abdominal pain that is worse on 1 side, or tubal rupture with hemorrhagic shock.^[4]^ As the patient always considered vaginal bleeding as menstruation, suspicion of pregnancy did not arise. This may be the major reason for the delayed diagnosis. Extrauterine pregnancy is always diagnosed in the first trimester of gestation, as most patients present with nonspecific complaints. Improved diagnostic and therapeutic methods have resulted in extrauterine pregnancy mostly paused to the 6th through 9th week.^[2]^ Abdominal computed tomography combined with transabdominal ultrasound was performed in this case for preoperative diagnosis. The hemoperitoneum and fetus in the abdominal cavity were visualized where the attached placenta was not accurately reported, and the diagnosis of abdominal pregnancy was suspected. Although diagnostic accuracy has increased over time and in modern practice, the majority of ectopic pregnancies are visualized on ultrasound scans before surgery and are often misdiagnosed and treated as abdominal pregnancies, which are proven to be advanced tubal pregnancies after laparotomy.^[5]^ Ultrasonographic criteria have been suggested for the diagnosis of an abdominal pregnancy, which includes the following: a fetus and gestational sac observed outside the uterine cavity, or the visualization of an abdominal or pelvic mass identifiable as a uterus but separated from the fetus, absence of the uterine wall between the bladder and the fetus, adherence of the fetus to an abdominal organ, and an abnormal location of the placenta outside the uterine cavity.^[6]^ The fetus in the second trimester slips out of the ruptured fallopian tube and leads to a large rupture and massive abdominal bleeding in a short period. Current evidence shows that specific diagnostic and therapeutic criteria for second-trimester tubal pregnancy are lacking owing to its rarity.^[7]^ Before proceeding with the operation, the surgical team meticulously evaluated the differential diagnoses, giving due consideration to primary abdominal pregnancy—a strikingly uncommon scenario where a fertilized ovum implants directly onto the peritoneal surfaces, mesentery, omentum, or other intra-abdominal structures, bypassing the typical route through the fallopian tubes. The rarity of this condition introduces distinctive complexities in both its clinical diagnosis and management, demanding heightened vigilance and expertise from the medical practitioners involved. Abdominal pregnancies are associated with a high risk of maternal morbidity and mortality. The management of early-stage abdominal pregnancy is primarily surgical, with laparotomy being the most common approach, especially in hemorrhagic cases.^[8]^ Laparotomy is typically favored over laparoscopic surgery in the management of suspected abdominal ectopic pregnancies due to the heightened risk of uncontrolled perioperative bleeding from the implantation site.^[9]^ The patient with a high suspicion of hemorrhage was emergently taken to the operating room. Consistent with this case, intraoperative reinfusion of autologous blood was possible through the blood collected during the operation. Caution should be exercised when considering autologous blood reinfusion in intraperitoneal pregnancy as the blood may be contaminated by substances from the ruptured digestive tract. Midline laparotomy is considered the preferred surgical approach in patients with life-threatening intra-abdominal bleeding and an unknown source of the large fetus. Tubal preservation was not suitable for use in the patient we reported as the fallopian tube accommodating the placental attachment had invaded and destroyed. In general, patients with ruptured ectopic pregnancies presenting with hypovolemic shock must be managed with urgent surgery,^[10]^ salpingectomy is a better choice for women with severely damaged fallopian tubes and large tubal pregnancies (>5 cm).^[11]^ Meanwhile, management options include drainage of the hemoperitoneum and replacement of blood and products. However, for treatment of abdominal pregnancy, the fetus should be delivered as soon as possible. There are 2 options for the management of the placenta: removal of the placenta or leaving the placenta in situ. Affecting the function of the viscera or forcing the placenta to divest may not result in serious bleeding, such as those located on the surface of the greater omentum, and partial viscera resection while removing the fetus. When the placenta is attached to important organs, such as the liver or large blood vessels, it can be placed in the abdominal cavity to avoid excessive bleeding. In most cases, the placenta is gradually absorbed with the possibility of abnormal coagulation during absorption.^[12]^ The issue of removing the placenta during surgery remains controversial. Recently, the treatment of retained placenta with methotrexate has been abandoned. Instead, a conservative approach involving the injection of potassium chloride (KCl) into the fetal heart under sonographic guidance has been suggested.^[13]^

This study is limited by its focus on a single case, which restricts the ability to generalize the findings. The presence of hemoperitoneum may have affected the accuracy of imaging diagnostics, leading to an initial misdiagnosis. Additionally, the treatment approach discussed is highly specific to this case and may not be applicable in all clinical scenarios, especially where resources are limited. The absence of established guidelines for second-trimester tubal pregnancies further limits the broader application of the study’s conclusions. These factors underscore the need for further research to enhance diagnostic and treatment protocols in similar cases.

## 4. Conclusion

In this report, we present a distinctive case of a second-trimester tubal pregnancy initially misdiagnosed as abdominal pregnancy. This case underscores the significance of precise diagnosis and prompt surgical intervention, which are crucial for improving maternal prognosis. It is imperative for healthcare professionals to develop highly specific diagnostic and therapeutic criteria for advanced tubal pregnancy. In light of the diverse treatment strategies required, it is critically important to consider the potential scenario of an abdominal pregnancy during presurgical evaluations. This ensures that all necessary precautions and considerations are taken to provide the most appropriate care and intervention for the patient. Additionally, educating the public on distinguishing abnormal uterine bleeding from menstrual bleeding can be an effective strategy to mitigate the occurrence of advanced ectopic pregnancies.

## Author contributions

**Conceptualization:** Zhiqiang Wang, Chun Zhang.

**Data curation:** Zhiqiang Wang.

**Formal analysis:** Zhiqiang Wang.

**Investigation:** Zhiqiang Wang.

**Methodology:** Zhiqiang Wang.

**Writing – original draft:** Zhiqiang Wang.

**Funding acquisition:** Chun Zhang.

**Supervision:** Chun Zhang.

**Validation:** Chun Zhang.

**Writing – review and editing:** Chun Zhang.
